# Evaluating the efficacy of a novel endometrial receptivity test in improving pregnancy outcomes for Chinese in vitro fertilization patients: a single-center retrospective study

**DOI:** 10.1186/s12884-026-09790-y

**Published:** 2026-08-10

**Authors:** Chunyan Ji, Xiaoyan Wang, Jinfang Yao, Xusheng Zhao

**Affiliations:** 1 Reproductive Center, Tianjin Aiwei Hospital, 138 Jintang Road, Hedong District, Tianjin, 300011 China; 2 Tanggu Maternity Hospital, Binhai New Area, Tianjin, 300011 China

**Keywords:** Advanced maternal age, Embryo transfer, Endometrium, In vitro fertilization, Pregnancy outcome

## Abstract

**Background:**

Current evidence on whether endometrial receptivity tests improve pregnancy outcomes among in vitro fertilization (IVF) patients remains conflicting and limited. This study aimed to evaluate the efficacy of HerRecepta, a novel transcriptomic endometrial receptivity test, in guiding personalized embryo transfer (pET) for Chinese IVF patients, with a focus on recurrent implantation failure (RIF) and clinical factors influencing the window of implantation (WOI).

**Methods:**

This retrospective study included 220 IVF patients who had undergone endometrial receptivity testing. Based on the receptivity test results, patients were stratified into the standard embryo transfer (ET) group or pET group. Pregnancy outcomes following frozen embryo transfer were then compared between groups.

**Results:**

Of the 220 patients, 124 (56.36%) had a receptive endometrium and underwent standard ET, while 96 (43.64%) had a non-receptive endometrium and underwent pET. Among the latter group, 69 (71.87%) were classified as pre-receptive. Advanced age (> 35 years, *P* = 0.036) and high anti-Müllerian hormone (AMH) levels (> 5 ng/mL, *P* < 0.001) were associated with a higher rate of non-receptivity. The overall clinical pregnancy rate (CPR) and live birth rate (LBR) were 46.50% and 32.11%, respectively. The pET group showed comparable outcomes to the standard ET group in CPR (46.36% vs. 46.67%, *P* = 0.966), and LBR (32.38% vs. 31.76%, *P* = 0.930). Similar results were observed in the RIF subgroup (124 patients: 79 in the standard ET group and 45 in the pET group).

**Conclusion:**

HerRecepta-guided pET yielded pregnancy and live birth rates comparable to those achieved with standard ET. Given the retrospective design and lack of a control group, these findings support the feasibility of this test rather than demonstrating its effectiveness or superiority, and further randomized trials are warranted.

## Introduction

Successful embryo implantation is a critical step in achieving pregnancy through assisted reproductive technologies (ART). Despite significant advances in in vitro fertilization (IVF) techniques [1], implantation failure, particularly recurrent implantation failure (RIF), remains a major barrier to successful treatment for many couples [2].

The window of implantation (WOI), a transient period when the endometrium is optimally receptive to embryos, is critical for implantation success. Traditionally, the timing of embryo transfer has relied on standard protocols that assume a uniform WOI. However, emerging evidence confirms significant individual variability in the WOI [3], and displacement is particularly common in patients with RIF [4]. This realization led to the development of various diagnostic tools designed to identify the optimal timing for embryo transfer on an individual basis.

One such tool that has gained considerable attention is the commercial Endometrial Receptivity Analysis (ERA) test [5, 6]. However, existing data on ERA’s efficacy in improving pregnancy outcomes are conflicting, with some studies reporting benefits [7–9] and others finding no advantage over standard transfer [10]. The conflicting evidence highlights the need for further investigation into the potential for optimizing this diagnostic method as well as defining the appropriate target population. Notably, data specific to Chinese IVF patients are limited, despite the high demand for ART in China, highlighting the need to understand the applicability and efficacy of endometrial receptivity testing in this population.

HerRecepta, a novel transcriptomic endometrial receptivity test, was constructed based on endometrial transcriptome and outcome data from over one thousand Chinese IVF patients. This single-center retrospective study aimed to evaluate HerRecepta’s efficacy in guiding personalized embryo transfer (pET) and explore clinical factors influencing WOI displacement. By doing so, this study provides evidence for optimizing personalized IVF treatment strategies in Chinese patients.

## Methods

We performed a single-center retrospective review including patients who agreed to undergo the HerRecepta testing between November 2019 and March 2024 at Tianjin Aiwei Hospital. To be included in the study, all patients underwent endometrial receptivity testing in strict accordance with the recommended timing specified in the test report when undergoing embryo transfer. Patients were excluded if they presented with reproductive tract organic lesions or malformations, concurrent endocrine or metabolic disorders, or uterine pathologies interfering with embryo implantation (e.g., endometrial polyps, uterine fibroids). This study was approved by the Institutional Review Board of Tianjin Aiwei Hospital. Signed informed consent was obtained from all participants. Patient characteristics including age, body mass index (BMI), anti-Müllerian hormone (AMH) level, infertility causes, history of pregnancy and embryo transfer, HerRecepta results, and pregnancy outcomes of the first embryo transfer cycle following receptivity testing were retrospectively extracted from the hospital electronic medical record system using de-identified data.

### Endometrial sampling and processing

Patients underwent HerRecepta testing under either hormonal replacement therapy (HRT) or natural cycles. In HRT cycles, exogenous estradiol was administered until endometrial thickness reached ≥ 7 mm with serum progesterone < 1.0 ng/mL, followed by progesterone supplementation and endometrial biopsy (50–70 mg) at 120 ± 3 h. Natural cycle biopsies were to occur after the luteinizing hormone surge (LH + 7) or human chorionic gonadotropin administration (hCG + 7). Samples were stored in RNAlater at 4 °C for ≥ 4 h prior to shipment to Hangzhou Yizhen Medical Laboratory.

### Endometrial receptivity testing and WOI recommendation

Total RNA was extracted from the endometrial specimens using a QIAsymphony RNA Kit (QIAGEN, 931636) according to the manufacturer’s protocol. RNA concentration was measured by spectrophotometry using a MultiSkan GO instrument (Thermo Fisher), and RNA integrity number was assessed using an Agilent 4200 TapeStation (Agilent). The library was constructed using VAHTS Universal V8 RNA-seq Library Prep Kit for Illumina (Vazyme, NR605) and sequenced on the NovaSeq 6000 platform (Illumina).

Over 240 endometrial receptivity-related gene expression values were processed, and the endometrial receptivity status was assessed by the HerRecepta computational predictor developed by HerAnova Biotechnology Co., LTD using a Python sklearn model (https://scikit-learn.org/stable/index.html). The receptivity results were reported as receptive or non-receptive. Non-receptive results included pre-receptive, early receptive, late receptive, and post-receptive. The optimal WOI was also recommended in the test reports.

### Receptivity test-guided embryo transfer and pregnancy outcome measures

Frozen embryo transfer (FET) was typically performed in the cycle after endometrial biopsy. A single cleavage-stage embryo on day 3 or a single blastocyst on day 5/6 was transferred. High-quality embryos were defined as follows: day 3 embryos required 7–10 cells with relatively equal cleavage and fragmentation rate less than 25%; blastocysts are defined as grade 3BB or higher according to the Gardner criteria. Embryo quality assessment was independently performed by two experienced embryologists using a uniform standard. Patients with a receptive endometrium underwent standard transfer (defined as standard ET group) in the cycle identical to the receptivity test cycle. In patients with a displaced implantation window or a non-receptive status, personalized embryo transfer (pET group) was performed based on the receptivity results’ recommendations.

Pregnancy outcome indicators, including pregnancy rate (PR), clinical pregnancy rate (CPR) and live birth rate (LBR, when available) were calculated. The pregnancy rate was defined as the proportion of women who were positive for serum β-human chorionic gonadotropin (β-hCG) (> 25 mIU/mL); CPR was defined as the proportion of women who were positive for β-hCG, and a gestational sac was visualized by vaginal ultrasound at the fifth week of pregnancy; LBR was defined as the proportion of live births at ≥ 22 weeks’ gestation.

### Statistical analysis

Normality was assessed using the Kolmogorov–Smirnov test. Continuous variables were presented as mean ± standard deviation (SD) or median (interquartile range, IQR) and compared using independent samples t-tests or Mann–Whitney U tests, respectively. Categorical variables are expressed as n (%) and analyzed using chi-squared or Fisher’s exact tests. Univariate and multivariate logistic regression models were constructed to identify factors associated with WOI displacement, and model fit was evaluated using the Hosmer–Lemeshow test. Post hoc power analysis (PASS 15.0) was performed for CPR and LBR (80% power, two-sided α = 0.05). *P* < 0.05 was considered significant. Analyses were conducted in R (version 4.4.0).

## Results

### Patient characteristics and overall pregnancy outcomes

Between November 2019 and March 2024, a total of 220 IVF patients completed HerRecepta testing and were enrolled in this study. Participant enrollment, grouping and analytical pathways are illustrated in (Fig. 1).

**Fig. 1:**
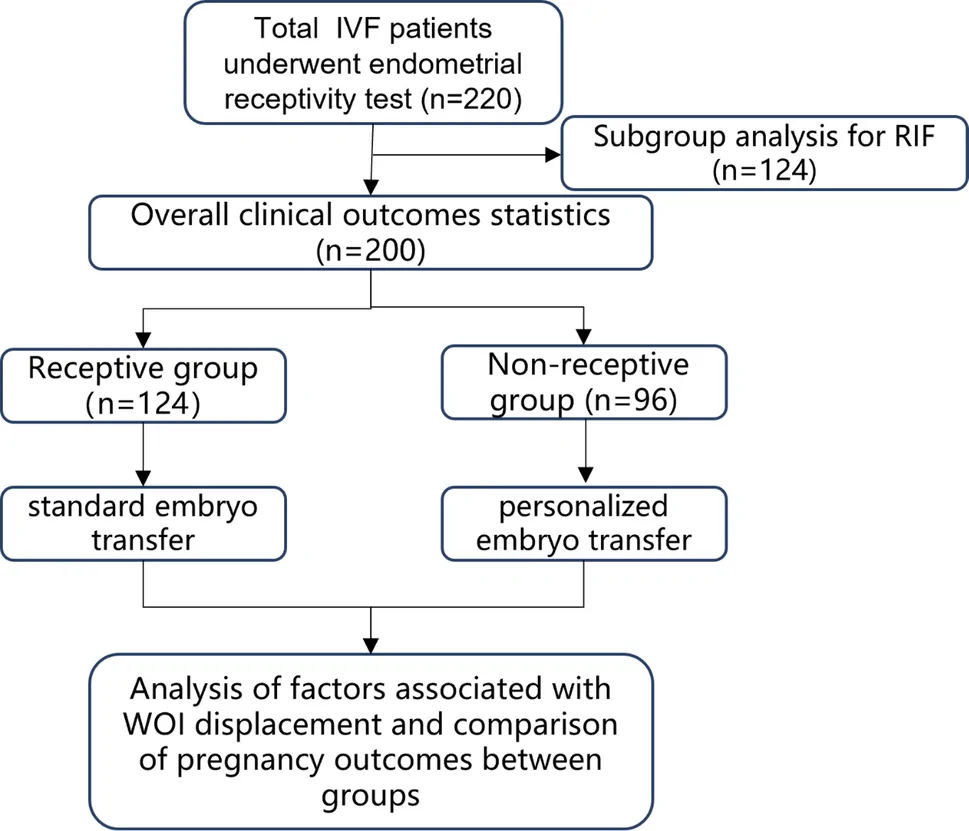
Flowchart of patient enrollment, grouping and analysis. Abbreviations: IVF, in vitro fertilization; RIF, recurrent implantation failure; WOI, window of implantation

Table 1 details the cohort’s mean age of 35.30 ± 4.30 years; among whom 66.36% (146/220) had ≥ 3 prior embryo transfer failures, 30.91% (68/220) had 1–2 failures, and 2.73% (6/220) had no prior failures (the latter were tested due to scarce viable frozen embryos). Tubal factors accounted for approximately 50% of infertility causes.

**Table 1:** Overview of patient characteristics

Characteristics	Results
Total patients, n	220
RIF (≥ 3 implantation failures), n (%)	146 (66.36)
1 or 2 prior implantation failures, n (%)	68 (30.91)
No implantation failures, n (%)	6 (2.72)
Prior implantation failures, Mean ± SD	3.40 ± 1.80
Age (years), mean ± SD	35.30 ± 4.30
BMI (kg/m^2^), median (Q_1_, Q_3_)	22.59 (20.72, 24.72)
AMH (ng/mL), median (Q_1_, Q_3_)	2.06 (1.24, 3.51)
Primary infertility, n (%)	126 (57.27)
Causes of infertility, n (%)	
Tubal factor	108 (49.09)
Ovulatory dysfunction	5 (2.27)
Endometriosis	17 (7.72)
Male factor	32 (14.55)
Combined male and female factor	32 (14.55)
Other female causes	18 (8.18)
Unexplained	6 (2.73)
Test results after biopsy, n (%)	
Receptive	124 (56.36)
Non-receptive	96 (43.64)
Pre-receptive	69 (71.87)
Early receptive	19 (19.79)
Late receptive	7 (7.29)
Post-receptive	1 (1.04)
Patients with reproductive outcomes post-test ^a^, n (%)	200 (90.90)
Transferred embryo type, n (%)	
Day 3/4	32 (16.00)
Day 5/6	168 (84.00)
Transferred high-quality embryo, n (%)	112 (56.00)
Overall pregnancy rate, n (%)	107 (53.50)
Overall clinical pregnancy rate, n (%)	93 (46.50)
Overall live birth rate ^b^, n (%)	61 (32.11)

a. Twenty patients were awaiting the embryo transfer cycle or awaiting the pregnancy results

b. Ten patients were still ongoing pregnancy at the time of data collection

*Abbreviations*: *BMI* body mass index, *AMH* anti-Müllerian hormone, *RIF* recurrent implantation failure, *SD* standard deviation, *Q*₁ first quartile, *Q*₃, third quartile

Of the 220 patients, 200 underwent FET after testing and had pregnancy outcomes documented by August 2024 (last follow-up). All patients underwent single-embryo transfer, with 84.0% receiving a day 5/6 blastocyst. Overall PR was 53.50% and CPR 46.50%. Of 93 clinical pregnancies, 61 resulted in live births, 22 in miscarriages, and 10 were ongoing at data cutoff (Table 1).

### Clinical factors affecting the WOI

According to HerRecepta results, 56.36% (124/220) of patients presented with a receptive endometrium, and 43.64% (96/220) were non-receptive; among non-receptive cases, 71.87% were pre-receptive, with only one post-receptive patient identified.

Binary logistic regression demonstrated that advanced maternal age (> 35 years, Odds ratio [OR] = 2.19, *P* = 0.012) and elevated AMH levels (OR = 1.19, *P* = 0.011) were independently associated with WOI displacement (Table 2). Natural cycle preparation tended to favor endometrial receptivity compared with HRT cycles, though the difference was not significant in multivariate analysis. The Hosmer–Lemeshow test confirmed good model fit (*P* = 0.56).

**Table 2:** Univariate and multivariate logistic regression analysis of factors associated with WOI displacement

Variables	Univariate					Multivariate				
	β	SE	Z	*P*	OR (95% CI)	β	SE	Z	*P*	OR (95% CI)
Age ≥ 35 years	0.58	0.28	2.09	**0.036**	1.78 (1.04–3.06)	0.78	0.31	2.51	**0.012**	2.19 (1.19–4.05)
BMI (kg/m^2^)	0.07	0.04	1.68	0.094	1.07 (0.99–1.16)	0.05	0.05	1.01	0.315	1.05 (0.96–1.15)
Secondary infertility	-0.00	0.27	-0.00	0.996	1.00 (0.58–1.71)	-0.04	0.31	-0.13	0.899	0.96 (0.53–1.76)
Natural cycle preparation	-1.36	0.66	-2.07	**0.039**	0.26 (0.07–0.93)	-1.23	0.67	-1.83	0.068	0.29 (0.08–1.09)
AMH (ng/mL)	0.13	0.06	2.34	**0.019**	1.14 (1.02–1.27)	0.17	0.07	2.54	**0.011**	1.19 (1.04–1.36)
Prior implantation failures	-0.01	0.08	-0.09	0.930	0.99 (0.86–1.15)	0.01	0.09	0.15	0.883	1.01 (0.86–1.20)
Endometrial thickness (mm)	-0.07	0.08	-0.97	0.334	0.93 (0.80–1.08)	-0.11	0.08	-1.32	0.188	0.89 (0.76–1.06)

*Abbreviations*: *WOI* window of implantation, *OR* odds ratio, *CI* confidence interval, *SE* standard error

Bold *P* values indicate statistically significant difference (*P*<0.05)

The distribution of endometrial receptivity across age strata is shown in Supplementary Table 1. The proportion of receptive cases gradually declined with advancing age, and patients over 35 years had a significantly lower receptivity rate than those ≤ 35 years (48.45% vs. 62.60%, *P* = 0.036; Table 3). For AMH, the WOI displacement rate was markedly higher in patients with AMH > 5.00 ng/mL than in those with AMH ≤ 5.00 ng/mL (74.07% vs. 39.38%, *P* < 0.001; Table 3).

**Table 3:** Endometrial receptivity status stratified by age and AMH level

Variables	Group	Receptive, *n* (%)	Non-receptive, *n* (%)	*P*
Age (years)	≤ 35	77 (62.60)	46 (37.40)	**0.036**
	> 35	47 (48.45)	50 (51.55)	
AMH (ng/mL)	≤ 5.00	117 (60.62)	76 (39.38)	**< 0.001**
	> 5.00	7 (25.93)	20 (74.07)	

*P-values were calculated by Chi-squared test

### Pregnancy outcome comparisons between receptive patients and non-receptive patients

We compared outcomes between receptive patients (standard ET group) and non-receptive patients (pET group). Baseline parameters were well balanced between groups (Table 4). No significant intergroup differences were found in PR (51.82% vs. 55.56%, *P* = 0.598), CPR (46.36% vs. 46.67%, *P* = 0.966), or LBR (32.38% vs. 31.76%, *P* = 0.930; Table 4). Between-group differences were − 3.74% (95% confidence interval [CI]: −17.61% to 10.13%) for PR, − 0.31% (95% CI: −14.22% to 13.60%) for CPR, and 0.62% (95% CI: −12.73% to 13.97%) for LBR. All confidence intervals included the null value, indicating comparable outcomes. Post hoc power analysis confirmed sufficient statistical power for CPR (99.8%) and LBR (98.6%), which substantially minimized the risk of type II error.

**Table 4:** Comparison of baseline characteristics and pregnancy outcomes between standard ET and pET groups

Characteristics	Standard ET (receptive)	pET (non-receptive)	*P*
Patients, n	124	96	
Age (years), mean ± SD	35.13 ± 4.21	35.61 ± 4.44	0.409
BMI (kg/m^2^), median (Q_1_, Q_3_)	22.34 (20.41, 24.43)	22.82 (21.21, 25.27)	0.079
AMH (ng/mL), median (Q_1_, Q_3_)	1.97 (1.21, 3.09)	2.16 (1.25, 3.92)	0.093
Prior implantation failures, mean ± SD	3.43 ± 1.64	3.41 ± 1.96	0.931
Primary infertility, n (%)	71 (57.25)	55 (57.29)	0.996
Patients with reproductive outcomes post-test ^a^, n (%)	110 (88.71)	90 (93.75)	0.197
Transferred high-quality embryo, n (%)	61 (55.45)	51 (56.67)	0.864
Pregnancy rate, n (%)	57 (51.82)	50 (55.56)	0.598
Clinical pregnancy rate, n (%)	51 (46.36)	42 (46.67)	0.966
Live birth rate ^b^, n (%)	34 (32.38)	27 (31.76)	0.930

a. Twenty patients were awaiting the embryo transfer cycle or awaiting the pregnancy results

b. Ten patients were still ongoing pregnancy at the time of data collection

*Abbreviations*: *ET* embryo transfer, *pET* personalized embryo transfer

Subgroup analysis of patients with RIF (defined as ≥ 3 prior transfer failures, age ≤ 40 years, according to Cimadomo et al. [11]; no recurrent miscarriage history) yielded results consistent with the overall cohort. No significant differences in PR (59.15% vs. 62.79%, *P* = 0.700), CPR (53.52% vs. 53.49%, *P* = 0.997), and LBR (40.30% vs. 32.50%, *P* = 0.420) were observed between standard ET and pET groups among RIF patients (Supplementary Table 2).

## Discussion

This single-center retrospective study of 220 Chinese IVF patients demonstrated that HerRecepta-guided pET resulted in pregnancy outcomes comparable to those achieved with standard ET, even among patients with RIF. Advanced maternal age and elevated AMH levels were identified as independent risk factors for WOI displacement. Collectively, these findings support the clinical feasibility of HerRecepta for WOI screening and personalized transfer guidance, though this study cannot confirm its superior performance or definitive therapeutic benefit.

Comparable clinical pregnancy and live birth rates were observed between the standard ET and pET groups, consistent with previous transcriptomic endometrial receptivity studies [12, 13]. In our RIF subgroup, pET achieved a live birth rate of 32.5%, whereas previous studies reported rates of 23% following standard ET in a meta-analysis [14] and 25% in a cohort study [15]. Although meta-analyses have reported relatively lower live birth rates with standard ET in RIF populations, cross-study comparisons should be interpreted cautiously given differences in patient characteristics, study design, and embryo culture protocols.

A key observation was the association between advanced maternal age (> 35 years) and reduced endometrial receptivity. Older patients in our cohort had a significantly lower receptivity rate, which corroborates studies linking reproductive aging to impaired uterine function [16, 17]. Elevated AMH levels (> 5.0 ng/mL) were strongly associated with WOI displacement in this study, a novel observation not documented in prior literature. Additionally, while univariate analysis suggested an association between artificial endometrial preparation and WOI displacement, this was not significant in multivariate analysis. Although Altmäe et al. [18] noted a potential negative effect of artificial cycles on endometrial receptivity, the association between preparation protocol and receptivity warrants further investigation.

This study has notable limitations. First, the study had a retrospective design and lacked a control group of patients who did not undergo HerRecepta testing. Second, the single-center derived samples may not fully represent the broader IVF population. Third, the present findings are primarily generalizable to Chinese IVF patients, and the applicability in non-Chinese populations remains unknown and requires further validation. Fourth, potential confounding factors such as embryo ploidy status, underlying endocrine disorders, and lifestyle factors were not fully matched or adjusted.

Considering the above limitations, we suggest prioritizing HerRecepta testing for high-risk subgroups including older patients and those with elevated AMH, to detect undiagnosed WOI displacement. Embryo transfer timed according to test findings may improve treatment outcomes for patients experiencing prior transfer failures.

Further large‑scale, multicenter prospective trials are essential to verify the clinical value and cost-effectiveness of HerRecepta among Chinese IVF patients. Stratified analyses based on age, AMH concentration and RIF severity are recommended to screen ideal recipients for endometrial receptivity testing. Moreover, subsequent work should focus on establishing multi‑dimensional assessment and intervention regimens for suboptimal endometrial receptivity.

## Conclusions

HerRecepta-guided pET yielded pregnancy outcomes comparable to those following standard ET. Due to the retrospective design and lack of a control group, the clinical utility of HerRecepta can only be suggested rather than confirmed. Further prospective studies are warranted. 

## Data Availability

The data used and/or analyzed during the current study is available from the corresponding author on reasonable request.

## Abbreviations

ART: Assisted reproductive technology
BMI: Body mass index
CI: Confidence interval
CPR: Clinical pregnancy rate
ERA: Endometrial receptivity analysis
ET: Embryo transfer
FET: Frozen embryo transfer
hCG: Human chorionic gonadotropin
HRT: Hormonal replacement therapy
IQR: Interquartile range
IVF: In vitro fertilization
LBR: Live birth rate
LH: Luteinizing hormone
OR: Odds ratio
pET: Personalized embryo transfer
PR: Pregnancy rate
Q₁: First quartile
Q₃: Third quartile
RIF: Recurrent implantation failure
WOI: Window of implantation
β-hCG: β-human chorionic gonadotropin
